# TuLeD (Tupían lexical database): introducing a database of a South American language family

**DOI:** 10.1007/s10579-020-09521-5

**Published:** 2021-01-13

**Authors:** Fabrício Ferraz Gerardi, Stanislav Reichert, Carolina Coelho Aragon

**Affiliations:** 1 Tübingen, Germany; 2 Joao Pessoa, Brazil

**Keywords:** Lexical database, Tupían, South American languages, Tupí-Guaraní, Linguistics

## Abstract

The last two decades witnessed a rapid growth of publicly accessible online language resources. This has allowed for valuable data on lesser known languages to become available. Such resources provide linguists with opportunities for advancing their research. Yet despite the proliferation of lexical and morphological databases, the ca. 456 languages spoken in South America are poorly represented, particularly the Tupían family, which is the largest on the continent. This paper therefore introduces and discusses TuLeD, a lexical database exclusively devoted to a South American language family. It provides a comprehensive list of lexical items presented in a unified transcription for all languages with cognacy assignment and relevant (cultural or linguistic) notes. One of the main goals of TuLeD is to become a full-fledged database and a benchmark for linguistic studies on South American languages in general and the Tupían family in particular.

## Introduction

Linguistic and ethnographic databases have served as a benchmark for a wide range of studies, and thus contributed to the understanding of both the prehistory of languages and the dynamics of language itself. They have allowed for the formulation of hypotheses and inferences about speakers of past languages, their culture (also material), their location, their migratory processes and their relation with other groups (Galucio 2010; Eriksen and Galucio 2014). Language data plays a significant role in ethnological studies (Walker et al. 2012; Berlin 1992; Berlin et al. 2013; Balée 2013) in general.

In response to the need for large quantities of tidily organized data and owing to the appearance of an open source software framework, the rising number of databases has immensely contributed to the progress of linguistic research since the last decade. Among the online databases one could mention: TransNewGuinea (Greenhill 2015), IELex (Dunn 2015), ASJP (Wichmann et al. 2018), ABVD (Greenhill et al. 2008), CHIRILA (Bowern 2016), LexiRumah (Kaiping and Klamer 2018) and NorthEuraLex (Dellert et al. 2019); others accounting for syntax, morphology or other language aspects, such as SAILS (Muysken et al. 2016), WOLD (Dryer and Haspelmath 2013), AfBo (Seifart 2013), and HG (Bowern et al. 2020).

The CLLD (Cross-Linguistic Linked Data) framework (Forkel et al. 2019) upon which most of the above mentioned databases are built, has allowed uniform access to and exchange of cross-linguistic data. This development goes hand in hand with the refinement of algorithms capable of identifying and extracting patterns from data. The standardized data format both within individual projects and across the various already published databases (Forkel et al. 2018; Rzymski et al. 2020; Wu et al. 2020) plays a fundamental role.

To our knowledge, among the available databases only CSD (Rankin et al. 2015) and SAILS (Muysken et al. 2016) deal with languages of the Americas so that the main bottleneck for TuLeD is the nearly total absence of lexical databases dedicated to South-American languages. The scarcity of available data is perhaps best explained by the fact that building up sizeable collections requires intensive manual labour and expert judgement for cognacy assignment, more easily found for well-studied languages (Jäger 2018).

The Tupían Lexical Database (TuLeD) here presented in its pre-release (v0.9) is the first online database exclusively devoted to a South-American language family. The database is open source^1^ and includes references to all consulted sources, including unpublished materials used in the data collection.

## Languages

**Fig. 1:**
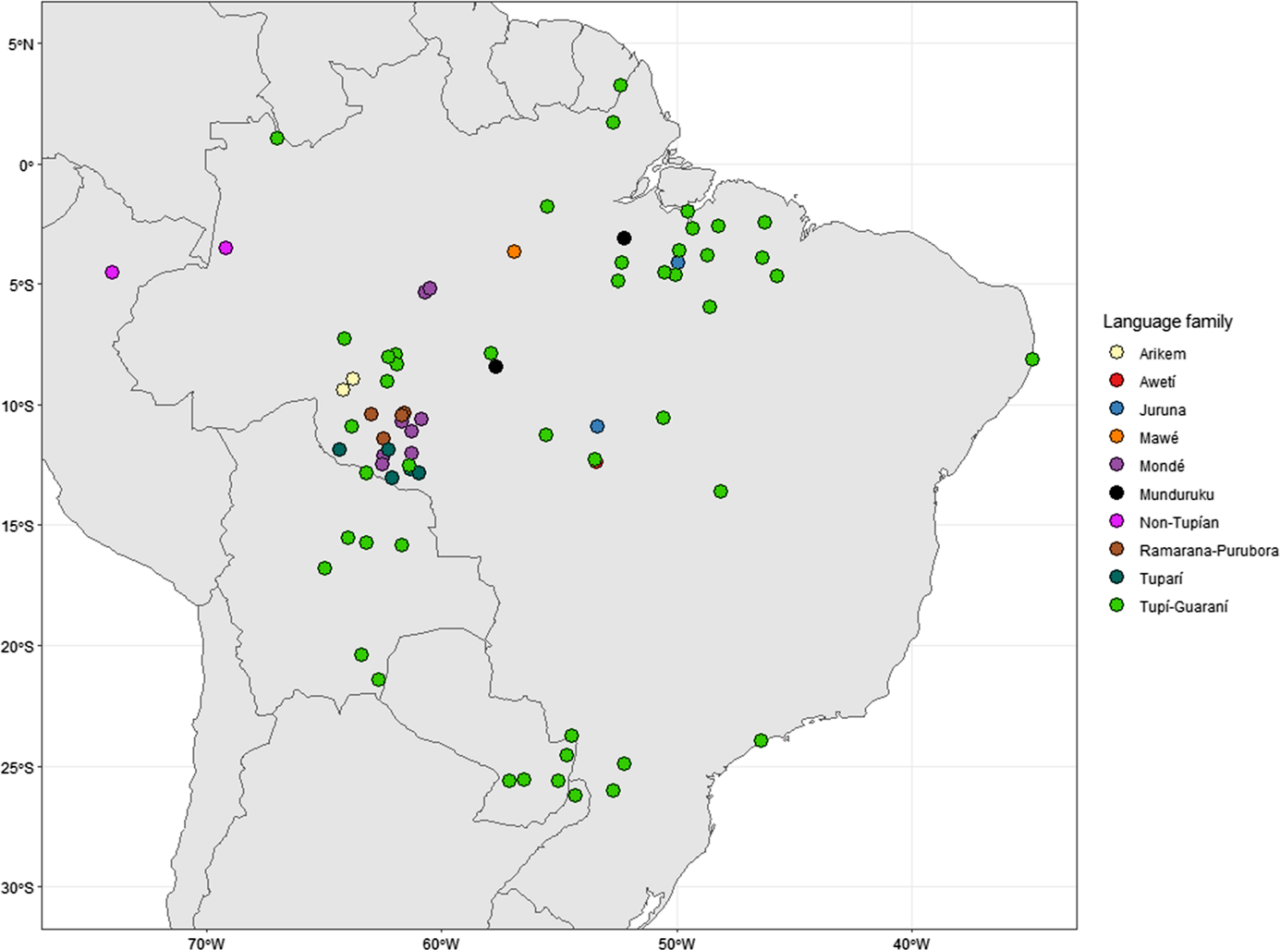
Map of languages in TuLed 0.9. Each Tupían subfamily is encoded by a different color. (Color figure online)

The seventy-four languages^2^ in TuLeD (see Fig. 1) belong to the Tupían family, the largest language family in South America. All subfamilies are represented in the dataset (Galucio et al. 2015; Rodrigues and Cabral 2012). We have also included extinct languages with different degrees of attestation, since they can be relevant for studying the geographical spread of Tupían languages and for the internal history of the family. A further criterion employed in order to distinguish language from dialect is the lexical distance measure between words for each language pair, as suggested by Wichmann (2020). The results obtained can be seen in Reichert and Gerardi (2021).

Tupi Austral, or ‘Língua Geral Paulista’ (which is a direct descendant of Tupinambá, like Nheengatu) was still spoken until the first half of the nineteenth century (Nobre 2011; Leite et al. 2013), and is mentioned in numerous historical sources, but only known through a list of words in Martius (2009) and a few other sources (Leite et al. 2013; Rodrigues 2010; Lagorio and Freire 2014), the main one anonymously compiled (Leite et al. 2013; d’Oliveira 1936). Similarly, Anambé of Ehrenreich (Ehrenreich 1895) is only known through a short list of ca. hundred words collected in the 19th century. The poorly attested Apapokuva, an extinct variety of Ava´-Guarani described by Nimuendajú (Nimuendajú 1914) (cf. Dietrich 2014), is also part of the dataset.

Two languages, for which there is insufficient information available, appear to belong to Ramarama-Puruborá group (Rodrigues and Cabral 2012; Gabas Jr. 2000): Ntogapíd (Itogapúk) is mentioned by Schultz (1925) who also provides a short wordlist (Nimuendajú 1955); Ramarama is mentioned with a wordlist by Lévi-Strauss (1950) and (Rondon and Horta Barbosa 1922). These have been included in Ramarana-Puruborá group due to the number of shared cognates between these languages and Karo and Puruborá.

**Fig. 2:**
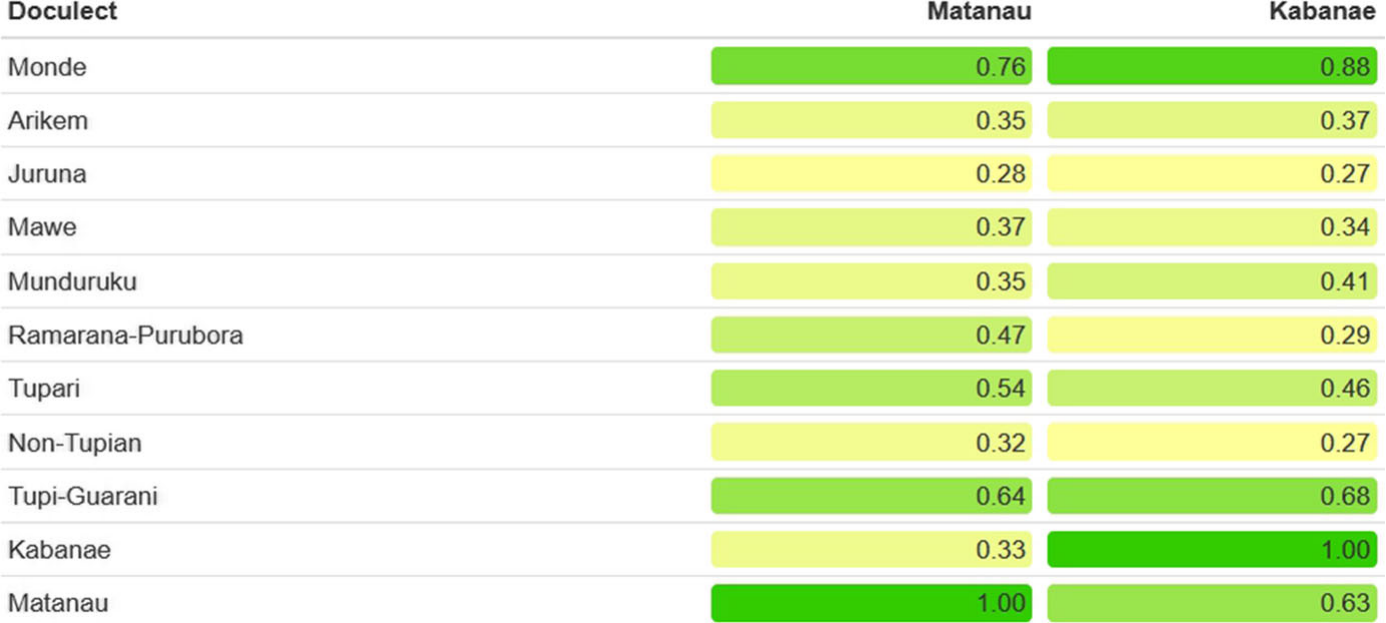
Amount, given in percentage, of cognates between Matanau and Kabanae, and each subfamily in the database

TuLeD is the first publication to include words from the languages Kabanae (Natterer 1829a) and Matanau (Natterer 1829b). Their inclusion is of a special interest as these languages almost certainly belong to the Mondé subfamily, given the similarity of the words collected by Natterer with words in other Mondé languages (see Fig. 2). This would, in turn, attest to the presence of Mondé groups on the banks of the Madeira River (da Silva and Costa 2014), quite apart from the historically attested Mondé languages^3^.

Little is known about Turiwara and Amanaye [(Loukotka 1968), pp. 110–113] except for the wordlists compiled by Nimuendajú (Nimuendajú 1914) and by a few mentions of these peoples (Nimuendaju 1948). The location of both tribes is known and despite the short wordlists, we can state with some degree of certainty which languages they are more closely related to (Rodrigues 1984). On the other hand, although extinct for centuries, Tupinambá and Old Guaraní are relatively well documented and have a large coverage—Tupinambá with a coverage of 97% of the concepts in the database.

As far as living languages are concerned, few things are worth mentioning. Within the Mondé languages, Gavião (Digüt/Ikólóéhj) and Zoró, are assigned the same Glottocode (Hammarström et al. 2020) and ISO-code (Eberhard et al. 2020), but there is enough evidence indicating that these are, in fact, two distinct languages (Moore 2005).

The picture is clearer in case of Kawahiv which is divided into two dialect groups: Northern and Southern. The former is formed by Parintintin, Juma, Jiahui and Tenharim, the latter by Urueuwauwau and Amondawa (others are not included in the database). Both these languages and their division seem to be consensual among specialists (Sampaio 1997, 2001; Aguilar 2015; Marçoli et al. 2018).

The database also includes Cocama-Cocamilla and Omagua two languages apparently of non Tupí-Guaraní origin, but whose lexicon is predominantly Tupí-Guaraní. The former has been said to be genetically unrelated to the Tupían languages despite the clearly Tupí-Guaraní lexicon (Cabral 1995; Michael 2014). The inclusion of the above mentioned extinct languages as well as Cocama-Cocamilla and Omagua is important in so far as they are extremely useful, among other venues of research, such as comparative work inferring contact and population movements.

Table 1 shows all of the languages in the database with the percentage of concepts for each language and their current version which, except for the extinct languages, is based on the Endangered Languages Project (ELP) (Languages Project 2020). Languages marked with a star (*) are not referenced in ELP, therefore their status is based on the authors’ knowledge and/or literature.

**Table 1:** Languages in the database with percentage of concepts in each of these and their respective status

Language	Coverage (%)	Status
Xipaya	86	Dormant
Juruna	74	Endangered
Karo (Arara)	77	Endangered
Puruborá	68	Critically endangered
Ntogapíd (Itogapúk)*	30	Extinct
Ramarama*	30	Extinct
Akuntsu	79	Critically endangered
Wayoró	75	Critically endangered
Makurap	72	Everely endangered
Mekens (Sakurabiat)	66	Critically endangered
Tuparí	80	Endangered
Mundurukú	99	Threatened
Kuruaya	70	Dormant
Cinta-Larga	12	Endangered
Gavião	74	Endangered
Aruá	52	Critically endangered
Matanau*	40	Extinct
Kabanae*	15	Extinct
Mondé	10	Dormant
Zoró	54	Endangered
Suruí-Paiter	82	Endangered
Karitiana	79	Endangered
Arikem*	56	Extinct
Sateré-Mawé	89	Threatened
Awetí	76	Endangered
Asurini Tocantins	68	Endangered
Parakanã	95	Threatened
Suruí	69	Endangered
Tapirapé (Apyãwa)	67	Endangered
Tembé	82	Severely endangered
Apiaká	72	Dormant
Guajajara	95	Vulnerable
Amondawa*	69	Threatened
Tenharim	73	Endangered
Jiahoi	28	Critically endangered
Parintintin*	93	Threatened
Juma	12	Critically endangered
Urueuwauwau	60	Endangered
Tupi do Machado (Wirafed)*	30	Extinct
Kayabí	63	Threatened
Asurini Xingu	76	Endangered
Araweté	61	Endangered
Kamayurá	81	Endangered
Anambé of Ehrenreich*	21	Extinct
Guajá	58	Endangered
Amanayé	28	Dormant
Zo’e	52	Endangered
Emerillon (Tekó)	88	Endangered
Wayampi	79	Threatened
(Urubu) Ka’apor	93	Endangered
Anambé	50	Nearly extinct
Turiwara*	28	Extinct
Avá-Canoeiro	64	Severely endangered
Tupinambá*	98	Extinct
Nheengatu	98	Endangered
Língua Geral Paulista (Tupi austral)*	5	Extinct
Yuki	61	Endangered
Guarayo	89	Threatened
Sirionó	79	Critically endangered
Warazu (Pauserna)	73	Critically endangered
Chiriguano	78	Endangered
Jorá*	17	Extinct
Mbyá	88	Vulnerable
Guarani Paraguay*	92	Official
Old Guaraní*	70	Extinct
Guayaki (Aché)	71	Severely endangered
Xetá	37	Critically endangered
Kaiowá	62	Vulnerable
Tapiete	85	Endangered
Chiripá	31	Endangered
Apapokuva of Nimuendajú*	30	extinct
Omagua	65	Critically endangered
Cocama-Cocamilla	72	Critically endangered

## The data

TuLeD in its actual pre-release version (0.9) includes 404 concepts. While databases vary considerably in their size: 40 items in ASJP (Wichmann et al. 2018) to 1310 in IDS (Key and Comrie 2015), the rationale determining the amount of concepts in TuLeD is to begin with the traditional Swadesh list (Swadesh 1950, 1952), the Leipzig-Jakarta list (Haspelmath and Tadmor 2009) and then to expand this list with items that are relevant to the Tupían culture (Heggarty 2010): cultivation, flora, fauna, food, housing, handicraft, hunting, kinship, spatial relations, social relations, and others (Rodrigues 2010; Galucio et al. 2015). The semantic fields according to which words are classified, are taken from World Loanword Database (WOLD) (Haspelmath and Tadmor 2009). Semantic fields in the database are given in Table 2.

**Table 2:** Presence of semantic fields for items in the dataset

Semantic field	Quantity (%)	Total
Agriculture and vegetation	30	7.44
Animals	80	19.85
Basic actions and technology	18	4.47
Body	60	14.89
Cognition	5	1.24
Clothing and grooming	5	1
Emotions and value	16	3.97
Food and drink	29	7.2
House	3	0.74
Kinship	31	7.69
Miscellaneous		
Function words	9	2.23
Motion	20	4.96
Physical world	25	6.2
Possession	3	0.74
Quantity	8	1.99
Religion and belief	1	0.25
Sense perception	19	4.71
Social and political relation	4	0.99
Spatial relation	19	4.71
Speech and language	5	1.24
Time	9	2.23
Warfare and hunting	5	1.24

Flora items have been shown to provide relevant information for language comparison and for inferring contact between and movements of populations (Balée 1994, 2013). As for the fauna, the basic ethnobiological terms in smaller societies with close link to nature tend to develop names for different species, often leaving gaps where one would expect more general terms (Berlin 1992; Atran 1993; Atran and Medin 2008). For this reason, some of the languages in the database lack, e.g. a general term for ‘monkey’ (Karitiana), while having names for individual species; many of the languages lack a hyperonym for the species of ‘ant’, having only words for single species. Since access to specific fauna and flora items is difficult—they are rarely if ever mentioned in the sources consulted—we are investigating ways to present them more thoroughly. Therefore, although the current amount of the diverse fauna and flora items in TuLeD is modest when compared to the overall number of concepts, the collection of relevant terms is ongoing and given high priority for the official release. It is important to note here that since TuLeD is not intended to be used exclusively for linguistic reconstruction or classification, we are not primarily guided by the argument according to which the size of the concept list would not necessarily improve classification (Holman et al. 2008).

The dataset also contains most of the *semantic primes* from (Wierzbicka 1996), and we made sure that all 56 oppositional concepts in Johansson (2017) are included. We consider these criteria of concept inclusion to be essential for search patterns or various inferences.

## Data collection

Besides the literature previously known to us, we are searching the repositories of Brazilian universities for new references, in particular the repositories of the university of Brasília (UnB) and the university of Campinas (UNICAMP), due to their long tradition of research in native Brazilian languages (master’s or doctoral theses from these universities comprise more than 17% of our bibliography). Another known source of research in native Brazilian languages consulted are the publications (bulletins and theses) of Emílio Goeldi Museum (13% of the sources). TuLeD has greatly benefited from these sources and from sources cited therein.

**Fig. 3:**
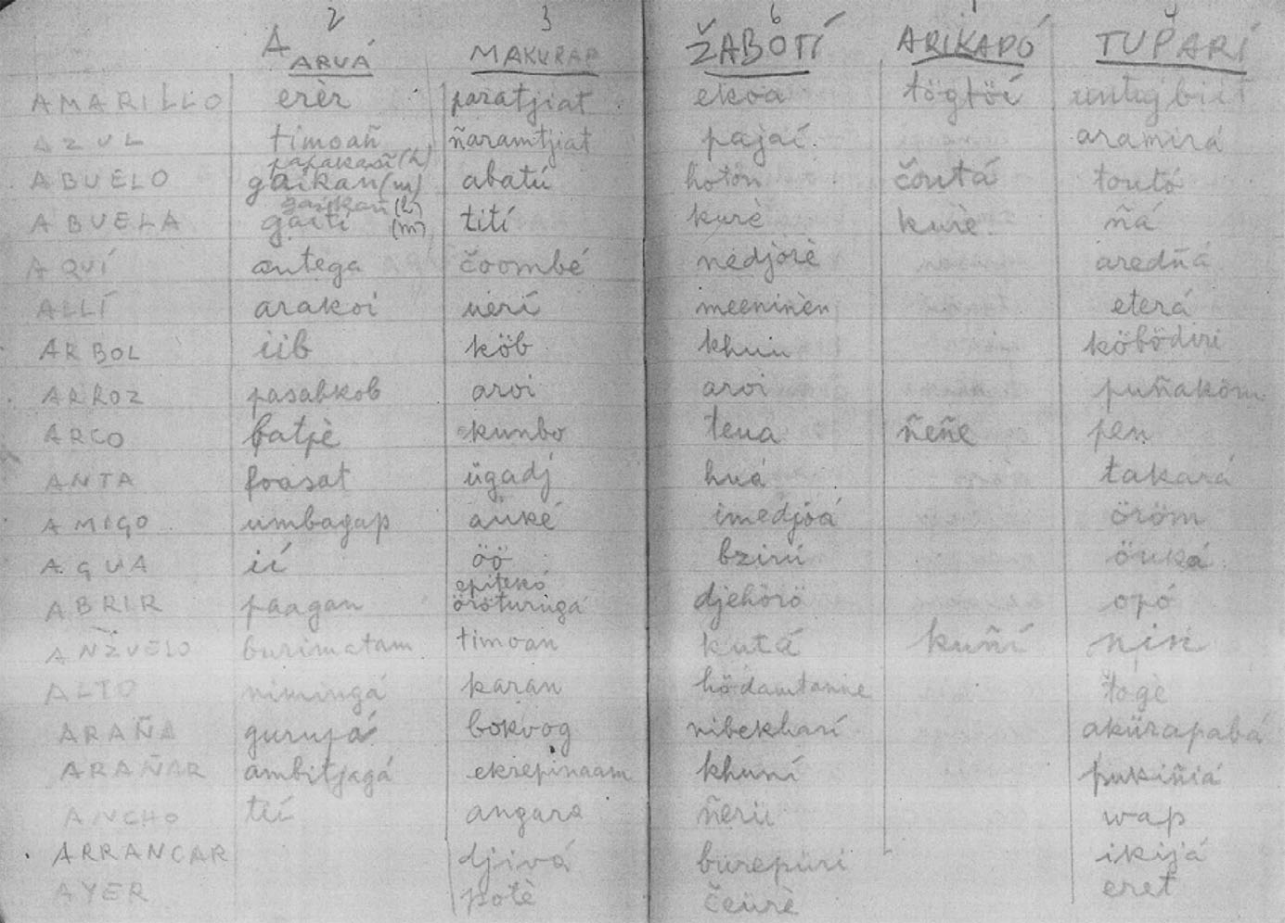
Page of Tibor Sekelj notebook containing words in five languages, three of them Tupían: Aruá, Makurap, and Tupari

**Fig. 4:**
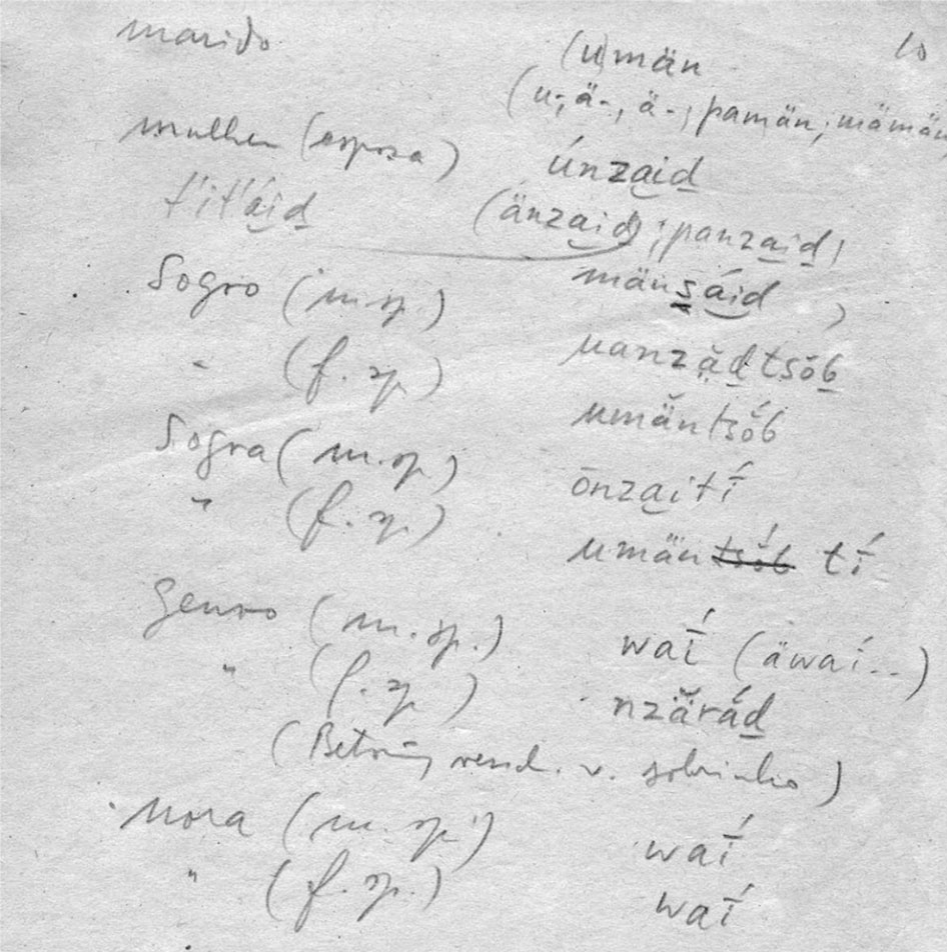
Original data collected by Franz Caspar in 1955 containing words in Aruá

**Fig. 5:**
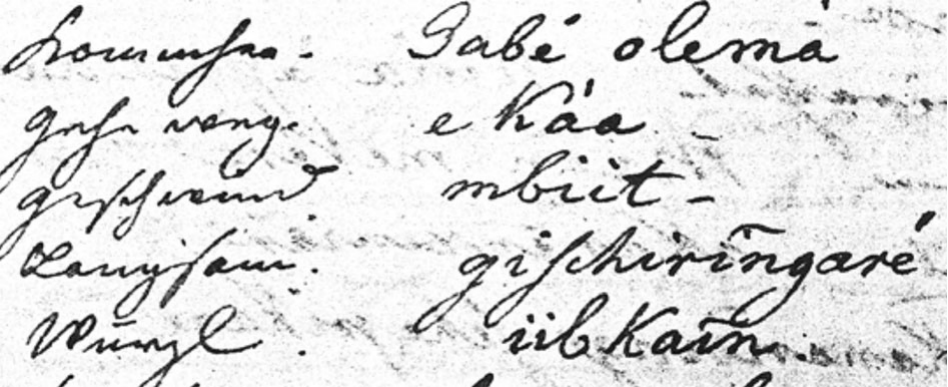
Fragment of Natterer’s Matanau–German wordlist (Natterer 1829b)

An evident shortcoming of the database stems from the poor quality of transcriptions provided by some of the sources collected by non-linguists. In this respect, Aruá is an illustrative case. Unpublished handwritten work accounts for most of the available data. Difficulties that arise when transcribing this type of data can be gleaned from Figs. 3, 4 and 5. Another illustrative examples are Kabanae (Natterer 1829a) and Matanau (Natterer 1829b), for which words have been compiled in 1830 by a native German speaker.

Poorly transcribed sources should not be used for tasks like phonological comparison or analyses involving distance methods. Yes despite the difficulties posed by the transcription, it is worth pointing out that it still allows, at least in the majority of cases, for cognate class assignment. This fact is illustrated in Table 3, where in spite of the transcription’s precision, cognate class can—most of the times—be clearly identified.

**Table 3:** Fragment of cognate class assignment from TuLeD, showing modern languages and one extinct language (Anambé of Ehrenreich). In spite of the probably imprecise transcription, cognates are recognizable

	Arrow	Bad/Evil	Big	Banana
Guajá			hu	pako
Ka’apor		ai		pako
Anambé of Ehrenreich	**wira**			**pareri**
Wayampí		ai		pako
Anambé (Carairi)	marara		uhu / **tuwihauhu**	**pariri**

### Additional features of TuLeD

**Fig. 6:**
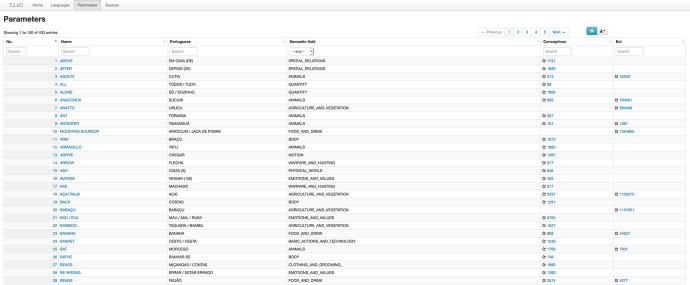
Screenshot of TuLeD’s Parameters environment

In the Parameters environment of the database, each of the 404 concepts is related to a semantic field taken from the WOLD (Haspelmath and Tadmor 2009), a link to the corresponding item in the Concepticon database (List et al. 2016a) which is a useful resource linking crosslinguistic lists. Flora and fauna items are each linked to the respective entries in the Encyclopedia of Life (EoL) (Parr et al. 2014)^4^, providing valuable information about the species in question. All this can be seen in Fig. 6.

## Transcription, segmentation, and alignment

All the data has been converted to the CLDF (cross-linguistic data format) using the CLTS (Cross-Linguistic Transcription Systems) (List et al. 2019) as a way of standardizing the data and making it easily shareable.

The tonal languages in the database have tones marked. In the case of Mondé languages, tones are marked according to the sources for each concept. Gavião has a more precise and complete marking of tones since most of the concepts have been retrieved from (Gavião 2019). The author is a native speaker who also provided us with concepts not present in the written work. For Mundurukú and Kuruaya, where available, the tones have been taken from (Picanço 2020). For languages without tones, the accents indicate where the stress falls.

**Fig. 7:**
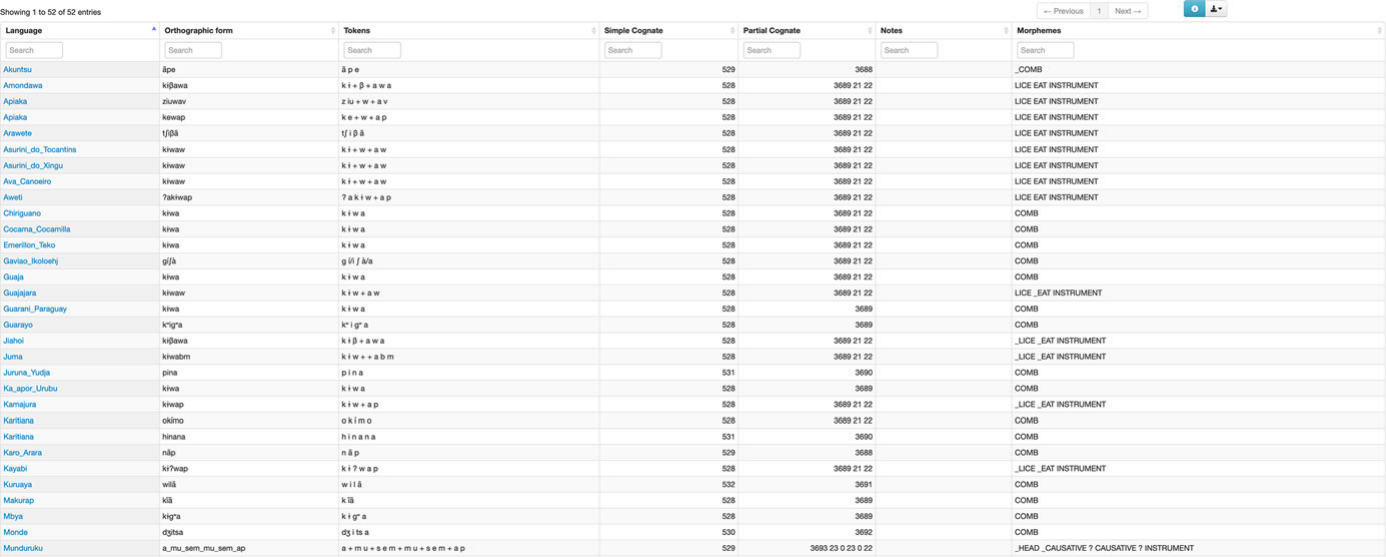
Screenshot of TuLeD’s Concepts environment showing the some of the words for the concept COMB

Transcription of each concept is given in the “orthographic form” column. This column is followed by the “tokens” column which contains segments. In this column, "tokens", when the etymology of the word is known, the segments of each part of the compound word are separated by a “+” sign. The meaning of each part of the compound can then be seen in the “morphemes” column where parts of the compound are separated by a single space. Figure 7 illustrates this using the concept COMB. The “notes” column generally includes information on borrowing, kinship terms, polysemy, and other relevant information. For the two languages Matanau and Kabanae, the “notes” column includes the original transcriptions of the words^5^.

The whole workflow described in this section closely follows (Wu et al. 2020).

### Simple cognacy, partial cognacy, and alignment

Simple and partial cognates had initially been automatically assigned using (List 2016; Hill and List 2017; List et al. 2016b; Wu et al. 2020), following automated detection. We have since manually improved simple and partial cognacy (expert judgement), and as of this writing (September 2020) 14% of entries have been manually improved. Cognacy assignment benefited from the following sources: (Galucio et al. 2015; Silva 2011; Kamaiurá 2012; Drude 2011; Rodrigues and Cabral 2012)) and is illustrated in Table 4. In order to visualize the data and align simple and partial cognates we have used the EDICTOR tool (List 2017). Partial cognacy is particularly useful due to the composite character of Tupían lexicon. They are useful in avoiding the transitivity issue, as illustrated in Table 5. The word for ‘cloud’ is presented in four languages and if cognate classes are based on the presence of *ɨwak-* ‘sky’, then Guajajara and Emerillon can be considered cognates. If instead, the presence of  ‘white’ is what defines the cognate class, then Suruí, Guajajara, and Emerillon are cognates, etc. Assigning numerical slots to each element of a compound (from left to right in Table 5) gives **245** (Suruí), **34** (Guajajara), **24** (Emerillon) and **13** (Asuriní Xingu). We have temporarily assigned cognate sets based on one of the units (mostly the head) of the compound. Thus, Suruí and Guajajara can be considered cognates due to the presence of **4**, Suruí and Emerillon due to **2** and **4**, Guajajara and Asuriní Xingu due to **3**. Asuriní Xingu, although cognate with Guajajara, cannot be considered a cognate with Suruí.

**Table 4:** Fragment of a cognate class assignment from TuLeD

Language	Concept	Phonetic form	Cognate class
Satere-Mawé	tapir	wewato	0
Avá-Canoeiro	tapir	tapir	1
Tupinambá	tapir		1
Sirionó	tapir	eãk^w^ãtoj	2
Chiriguano	tapir	mboréwi	3
Mbyá	tapir	mbore	3

Partial cognates are being assigned to each concept at a slower pace. Cognates are assigned according to the number of elements in the compound, which are separated by a dash (−), while cognate classes are separated by a single whitespace character. This is illustrated in Table 5, showing the word for ‘cloud’ and its cognate classes in some of the languages:

**Table 5:** The word for ‘cloud’ in four TG languages. Corresponding elements of the compounds occupy the same slot

	1	2	3	4	5
Suruí					ron
Guajajára					
Emerillon		arata			
Asuriní Xingu					

**Fig. 8:**
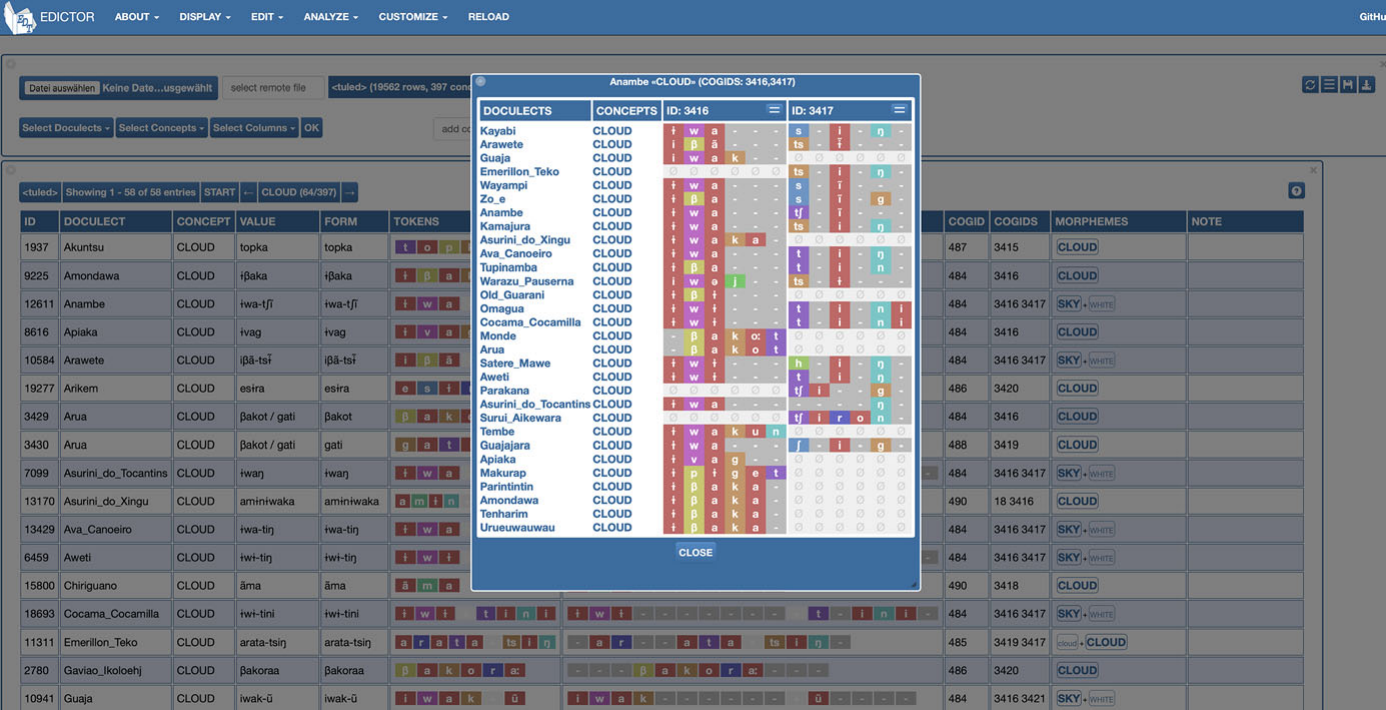
Screenshot of EDICTOR’s GUI available online at http://lingulist.de/edictor/

The use of EDICTOR for automatic alignment is useful but requires expert knowledge. Besides offering an initial alignment that saves time, it also provides good visualization for manual alignment improvement and cognacy correction if necessary. Figure 8 illustrates the way data is displayed and handled by the EDICTOR.

## Future challenges and outlook

This paper introduced the pre-release version of the lexical database exclusively dedicated to a South American language family. TuLeD has already proven its utility in the field of historical linguistics supporting a novel classification of Tupí-Guaraní languages (Ferraz Gerardi and Reichert 2021) based on a subset of the data. The results suggest promising new venues to apply the database, e.g. to provide the much needed data for further research.

Data expansion, specifically the addition of fauna and flora items, goes hand in hand with the refinement of simple cognacy and the assignment of partial cognacy, and requires correction (mainly the unification of the transcription across the sources) on a constant basis. The case of Tupían languages illustrates the need to combine the expertise of the researchers based on insights from multiple disciplines with the evolving computational approaches called for in Wu et al. (2020).

TuLeD is the first available part of TuLaR (Tupían Language Resources), which will include syntactical and typological data. We also plan to expand TuLeD without losing sight of the possibility of integrating it with still evolving (computational) tools.

TuLeD is a project that is being constantly updated and expanded. We expect it to become a benchmark for work on the Tupían family. Meanwhile we face several challenges of varying difficulty, ranging from data correction and improvement of simple and partial cognacy assignment to the inclusion of other relevant features and linking the entries to relevant online databases as described above.

## A list of semantic fields and concepts

Agriculture and vegetation:

açaí palm, anatto, bamboo, branch, bush, cará root, cocoa, corn, flower, genipa, grass, leaf, manioc, papaya, peach palm, peanut, root, seed, shell/bark, thorn, timbo liana, tobacco, tree, tucuma palm.

Animals:

anaconda, ant, anteater, armadillo, bat, bird, butterfly, capuchinmonkey, capybara, chameleon, cicada, coati, cockroach, crab, cricket (zool), curassow, deer, electriceel, firefly / glowworm,,fish, flea / chigger, fly (n), frog, gnat/pium, guan/jacu, hawk, hedgehog, hen/chicken, howlermonkey, hummingbird, jacaré/caiman/crocodile, jaguar, kingfisher, largeant (tocandira), large mandi fish, lizard, louse, macaco preto, macaw, monkey, mosquito, opossum, owl, paca, pacufish, parrot, peccary (collared), peccary (white-lipped). piranha, rat, scorpion, sloth, snail, snake, spider, squirrel, stingray, surubim fish, tapir, tayra, termite, tick, tinamou, toucan, trahirafish, turtle, vulture, wasp, wildcat, wilddog, woodpecker, worm, bee, dog, nest.

Basic actions and technology:

basket, break, cut, do/make, draw/paint, dry, hit, knife, pierce, rope, sweep, tie, untie, wash.

Body:

arm, back, bathe, beard, belly, bite, blood, bone, breast, breathe, bury, claw, defecate, die, ear, eye, face, feather, finger, foot, hand, hair, head, heal, heart, horn, kill, knee, leg, liver, liver, medicine, moustache, mouth, nail/claw, neck, nose, penis, saliva, sick/ill, skin, sleep, snore, stand, stomach, strong, tail, testicles, throat, tired, tongue, tooth, urinate, vein, vomit, wing, wing (2).

Clothing and grooming:

comb, cotton, dress up.

Cognition:

because, feel, know, learn, teach, think1

Emotions and values:

be wrong, cry, fear/be afraid, good//well, happy, laugh, pain/hurt, play, play (2) (cause to jump), sad(ness), scare, ugly, want.

Food and drink:

banana, beans, Brazil nut, cashew, drink (v), eat, egg, fat/grease, flesh/meat, flour, food, fruit, pepper, pineapple, porridge, pumpkin, raw, ripe, salt, suck, sweet potato.

Kinship:

boy, brother, father, girl, grand father, husband, man, mother, mother-in-law (of men), mother-in-law (of women), person/human being/someone, sister, son, uncle (MoBr), we (excl), we (incl), wife, woman, you (sg).

Miscellaneous function words:

here, not, other/some, same, that, this, what, who.

Motion:

arrive, blow, canoe, come, motion, enter, fall, fly, go, go up, move, path/way, return/come back, run, send, swim, walk.

Physical world:

ash, burn (intr), burn (tr), cloud, earth/land, fire, firewood, lake, moon, mountain, mould, rain, river, sand, sky, smoke, star, stone, sun, thunder, water, wind.

Quantity:

all/every, four, full, many, more, one, part, three, two.

Sense and perception:

black, blue, cold, dirty, dry (state), green, hear/listen, heavy, hot, look at, red, see, sharpen, sour/acid, sweet, wet, white, yellow.

Speech and language:

name, say, speak, tell/narrate, word.

Spatial relations:

above, after, before, big, far, flat, gather, grow, hide, hole, inside, lay down, near, put, round, side, sit, small, thick, under.

Time:

day, new, night, now, old, terminate/finish, tomorrow.

Warfare and hunting:

arrow, axe, bow, hunt.

## Footnotes

1 The data is available under Attribution-ShareAlike 4.0 International (CC BY-SA 4.0) license, and can be shared, copied, adapted and distributed as long as it is cited. The database itself is available online at: https://tuled.org/.

2 As pointed out by an anonymous reviewer, there is indeed an issue with the term language in contrast to dialects. One could be skeptical regarding, for example, the languages of the Mondé subfamily, the Kawahiv subfamily, or Asuriní do Tocantins and Parakanã. We follow the literature consulted, which is up-to-date, as can be seen from the resources in the database, and additionally provide ISO and Glotto-codes when available.

3 The locations in our map correspond to the locations of languages with similar names given by Nimuendajú in his map (do Patrimônio Histórico e Artístico Nacional 2017).

4 Available online at: https://eol.org/.

5 For Matanau and Kabanae we are working on making the transcriptions from the original documents visible online for each of the words. This feature will be available in version 1.0 of the database.
